# Multiple skin tumors in a patient treated with orelabrutinib for mantle-cell lymphoma: Case report

**DOI:** 10.1097/MD.0000000000046417

**Published:** 2025-12-12

**Authors:** Xuefeng Fu, Yuxi Zhang, Lei Zeng, Qiying Zhang, Xuefeng Fu

**Affiliations:** a Department of Dermatology, Jinhua Municipal Central Hospital Medical Group, Jinhua, Zhejiang Province, China; b Affiliated Jinhua Hospital, Zhejiang University School of Medicine, Jinhua, Zhejiang Province, China.

**Keywords:** basal cell carcinoma, case report, mantle-cell lymphoma, orelabrutinib, skin squamous cell carcinoma

## Abstract

**Rationale:**

Mantle-cell lymphoma (MCL) is an aggressive B-cell non-Hodgkin lymphoma. Bruton tyrosine kinase inhibitors (BTKi) significantly improve the prognosis of MCL, but their long-term use may induce immunosuppression-related complications, including secondary malignancies. First-generation BTKi such as Ibrutinib have been reported to be associated with the development of skin cancer.

**Patient concerns:**

A 77-year-old male patient with MCL treated with the next-generation highly selective BTKi Orelabrutinib (150mg/ day) developed multiple different types of skin tumors.

**Diagnoses:**

Skin pathological examination confirmed basal cell carcinoma of the nose, squamous cell carcinoma, actinic keratosis and seborrheic keratosis of the chest skin.

**Interventions:**

Orelabrutinib was suspended and switched to chemotherapy. The nasal and chest skin tumors were resected, the nasal wound was repaired with adjacent flap, and the chest wound was repaired with skin grafting.

**Outcomes:**

The wound recovered well after the operation, but the patient died of multiple organ failure due to pulmonary infection 5 months later.

**Lessons:**

Orelabrutinib may be associated with multiple skin tumors, and the mechanism may be through complex immune dysregulation. It is recommended that patients receiving long-term BTKi therapy undergo regular dermatological examinations.

## 1. Introduction

Mantle-cell lymphoma (MCL) is a type of B-cell non-Hodgkin lymphoma that is usually treated with immunochemotherapy and is prone to relapse after remission.^[1]^ In recent years, Bruton tyrosine kinase inhibitors (BTKi) have significantly improved the prognosis of MCL, but they may lead to immunosuppression-related complications, including secondary malignancies.^[2–6]^ First-generation BTK inhibitors such as ibrutinib have been reported to be highly associated with nonmelanoma skin cancer (NMSC).^[7]^ However, there were no relevant reports on skin tumors of Orelabrutinib, a new-generation of highly selective BTK inhibitor. We observed a rare case of multiple skin tumors (basal cell carcinoma [BCC], squamous cell carcinoma [SCC], AK, and seborrheic keratosis [SK]) that developed during treatment of MCL with Orebrutinib.

## 2. Case presentation

A 77-year-old male patient was admitted to the dermatology clinic for treatment with “brown spots on the nose for 1 year and a lump on the chest for 3 months”in May 2023. In March 2021, the patient was diagnosed with MCL (blastocyst M1) due to cervical lymph node enlargement. In July 2021, treatment with oral Orelabrutinib (150 mg per day) was started.^[8]^ His condition was stable while on medication. In April 2022, the patient suddenly found a brown spot on his nose without obvious discomfort and did not receive special treatment. Then, in February 2023, a lump was noted on the patient’s chest that was elevated above the skin. During the 3 months before coming to the dermatology clinic, the chest lump gradually increased without obvious pain or other discomfort. No history of lymphoma or skin cancer was found in the patient’s family. And the patient’s previous physicians did not detect significant changes in skin color, texture, or other changes during his MCL. The patient also denied any previous skin complaints such as dermatitis, eczema, injury, or pruritus. The patient had an ordinary indoor job, had no long-term history of sun exposure, or occupational UV exposure, and also had no history of taking photosensitive medications.

**Figure 1.**
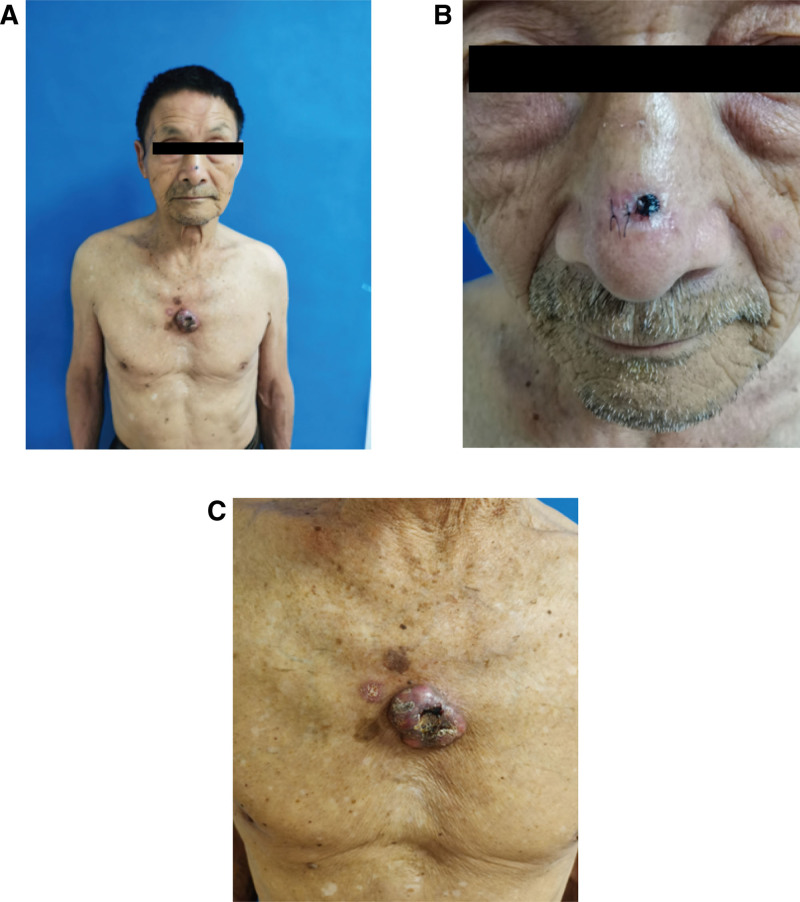
Preoperative photograph. (A) Overall upper body image of the patient showing a nasal tip mass and anterior chest mass. (B) Nasal mass with surgical sutures left by pathology. (C) Large mass on the chest, and abnormal plaques around the mass.

**Figure 2.**
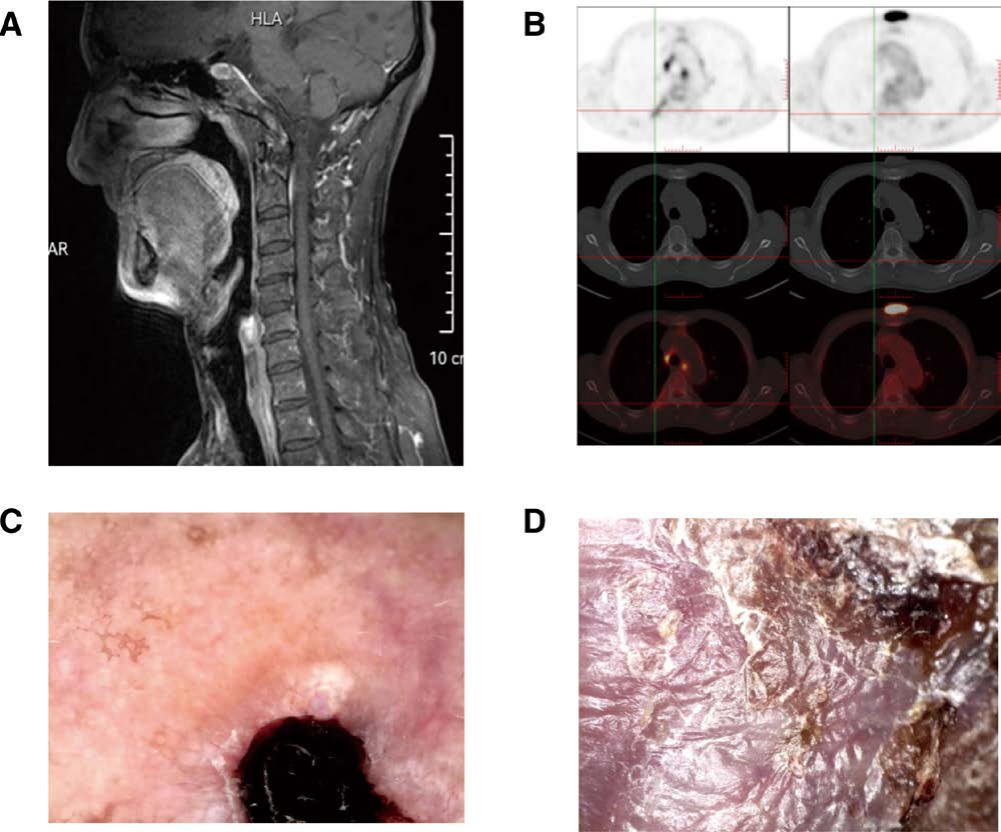
(A) Longitudinal nasopharyngeal MR scan: a slight thickening of the skin at the tip of the nose (B) Chest transverse PET-CT scan: anterior chest wall occupying with increased FDG metabolism (SUV max = 11.4) (C) Dermoscopic image of the nose: a well-defined blue-gray mass on nose with slender, branched blood vessels. (D) Dermoscopic image of the chest: a white or yellow unstructured area on a red background.

**Figure 3.**
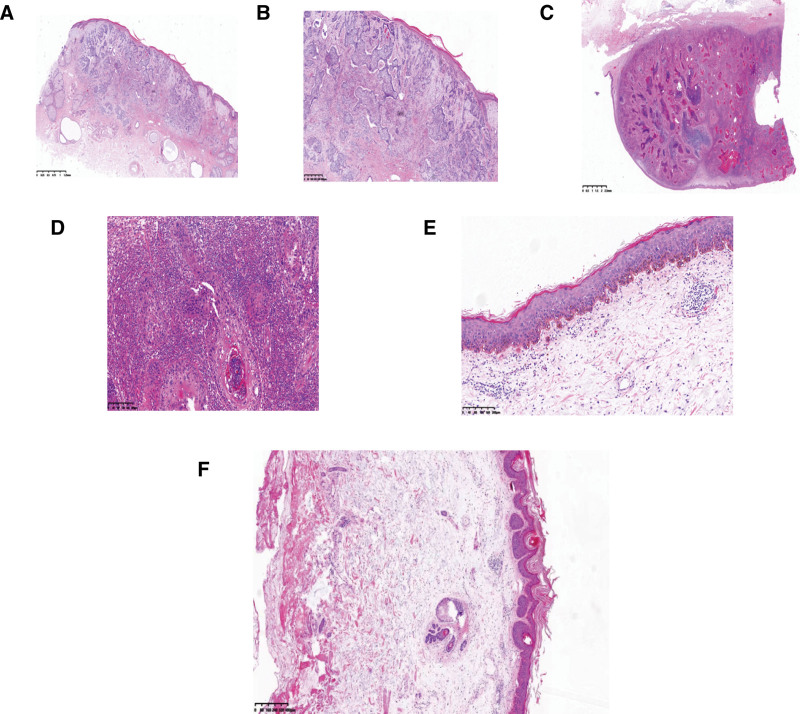
Histopathology, H&E staining. (A) A palisade arrangement of basal-like cells (nose), magnification × 20. (B) a palisade arrangement of basal-like cells (nose), magnification × 40. (C) Cells were arranged in nests, the cell atypia was obvious, and the formation of keratinized beads was visible (chest), magnification × 20. (D) Cells were arranged in nests, the cell atypia was obvious, and the formation of keratinized beads was visible (chest), magnification × 40. (E) Hyperkeratosis in the epidermis, hyper acanthosis, and dyskeratosis cells (dark erythema at the margin of the thoracic mass), magnification × 20. (F) Hyperkeratosis, spinous hypertrophy, false horn cyst (Brown spots along the border of the thoracic mass), magnification × 20.

Physical examination: The patient has type Ⅲ skin in the Fitzpatrick skin classification. There was a brown spot about 1 × 1 centimeter in size on the nose, with a little erosion. A lump about 4 × 3.3 centimeters in size was found on the chest, which was higher than the skin around, with a little ulceration, and dark erythema and brown spots were seen at the edge (Fig. 1A–C). Auxiliary examination: Blood routine showed white blood cells 4.43 × 10^9^/L, hemoglobin 120 g/L, platelets 153 × 10^9^/L. Nasopharyngeal Magnetic Resonance showed a slight thickening of the skin at the tip of the nose (Fig. 2A). Positron emission tomography/computed tomography (PET-CT) showed anterior chest walls occupying with increased FDG metabolism (SUV max = 11.4) (Fig. 2B), and multiple lymph nodes in the axilla, mediastinum, hilum, and posterior peritoneum on both sides. Dermoscopy shows a well-defined blue-gray mass on the nose with slender, branched blood vessels (Fig. 2C), and a white or yellow unstructured area on a red background (chest) (Fig. 2D). Skin biopsy showed a palisade arrangement of basal-like cells (nose), which was diagnosed as BCC (Fig. 3A and B). While the tumor cells were arranged in nests, the cell atypia was obvious, and the formation of keratinized beads was visible (chest), which confirmed the diagnosis of skin SCC (Fig. 3C and D). The biopsy results of dark erythema at the margin of the thoracic mass showed hyperkeratosis in the epidermis, hyper acanthuses, and dyskeratosis cells, which were consistent with AK (Fig. 3E). Brown spots along the border of the thoracic mass are consistent with SK (Fig. 3F). Examination of lymphocyte subsets at the time of diagnosis of MCL, initiation of Orelabrutinib and discovery of nasal spots in the patient is presented in Table 1.

**Table 1:** The lymphocyte subsets of 2021.3, when diagnosis of MCL, and 2021.7, when initiation of Orelabrutinib, and 2022.4, when discovery of nasal spots.

Lymphocyte subsets of 3 time points							
Time	T cells % (CD3+)	Th cells % (CD3 + CD4+)	Ts cells % (CD3 + CD8+)	Th/Ts cell (CD4+/CD8+)	Td cells % (CD4 + CD8+)	B cells % (CD19+)	NK cells % (CD3-CD16 + CD56+)
2021.3	23.96	4	19.57	4.08	4.8	0.09	70.31
2021.7	41.26	24.32	16.82	1.45	0.24	48.44	9.95
2022.4	87.01	56.48	30.43	1.86	0.38	1.71	10.35

MCL = mantle-cell lymphoma, NK cells = nature killer cells, Td cells = delayed reaction T cells, Th cells = helper T cells, Ts cells = T suppressor cells/regulatory T cells.

**Figure 4.**
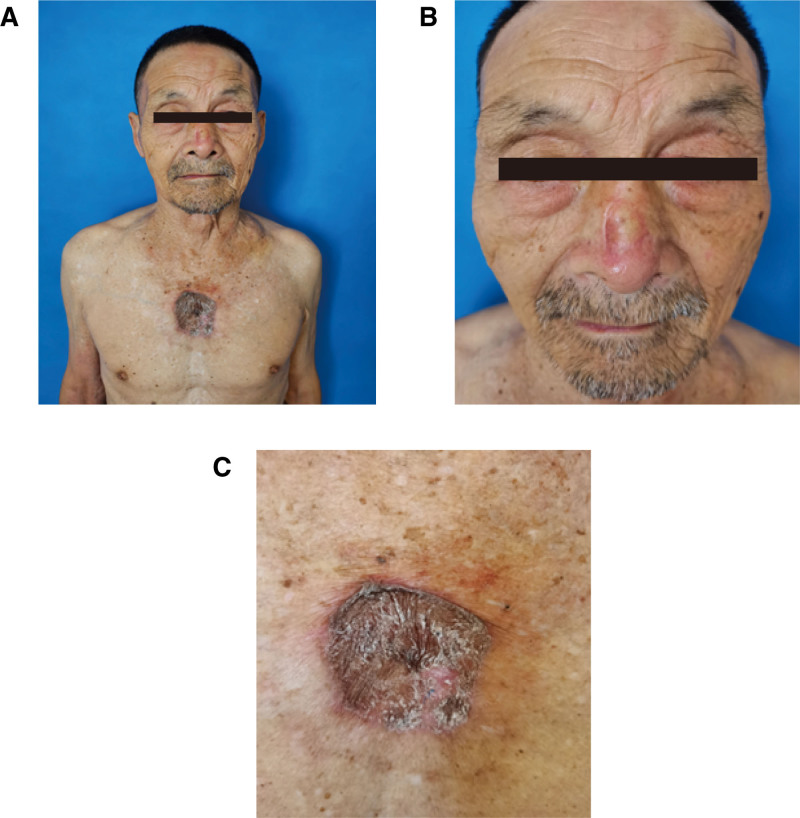
Photograph 1 mo after surgery. (A) Overall upper body images of the patient show postoperative recovery from the nasal tip mass and the anterior chest mass. (B) A month after nose surgery. (C) One month after chest skin grafting.

After discussion with the patient and his family, the patient underwent skin tumor resection (nose) and adjacent flap repair, while skin tumor resection (chest) and skin grafting were under general anesthesia. The postoperative histopathology confirmed negative incision margin. The patient recovered well after surgery without significant discomfort (Fig. 4A–C). Considering that the skin tumor might be related to Orelabrutinib treatment, Orelabrutinib treatment was suspended before surgery and chemotherapy was given after surgery. Five months after surgery, the patient died of multiple organ failure due to a lung infection (relevant imaging suggested no tumor metastasis). The patient’s family consented to the publication of the patient’s clinical and graphic details.

## 3. Discussion

BCC and SCC are common skin malignancies that usually occur as a single disease, the main risk factor is sun exposure.^[9]^ Ultraviolet light exposure is considered a risk factor for SK, which is therefore prone to appear on the face and upper chest.^[10]^ AK is a pre-skin cancer that is closely associated with sun exposure and immunosuppression.^[11]^ The patient in this case complicated with 4 kinds of skin benign and malignant tumors at the same time, distribution in the nose the exposure area and chest the nonexposure site, and the patient lived at 29° North latitude and denied a long history of sun exposure, which was clinically rare.

MCL is a rare B-cell non-Hodgkin lymphoma, which can lead to immunosuppression.^[12,13]^ BTKi have shown promising efficacy in the treatment of MCL.^[2]^ BTK is one of the key enzymes of the B-cell receptor (BCR) pathway that affects the growth, proliferation, adhesion and survival of B cells. Then inhibition of BTK has potential deleterious effects on immune responses, such as the development of opportunistic infections, including invasive fungal infections, in patients receiving Ibrutinib.^[6,14]^

The lymphocyte data of the patient in this case present a complex picture. The drastic reduction of B cells (CD19+), from 48.44% at the start of treatment to 1.71% at the time of skin tumor diagnosis, demonstrated the potent targeting of Orelabrutinib in controlling MCL, while a significant increase in total T cell (CD3+) and helper T cell (CD4+) numbers was observed (Table 1). Successful suppression of the B-cell lineage with recovery of the T-cell population constitutes a key paradox -the effective control of primary malignancies coincides with the emergence of secondary skin cancers, underscoring that deep control of the pathogenic cell lineage may inadvertently disrupt the balance of the integrated immune network when targeted agents are used in B-cell malignancies. Effective antitumor immunity depends not only on the number of immune cells, but also on their function and balance within the tumor microenvironment.

Given the important role of B cells in the process of antigen presentation to T cells, B cells depletion might change the balance of immune steady-state and cytokines.^[15]^ BTKi reduce IL-2-induced T-cell kinase (ITK) activation and phosphorylation of the T-cell receptor (TCR) and inhibit Janus kinase-signal transducer and activator of transcription (JAK-STAT) signaling associated with T-cell activation.^[16,17]^ Despite recovery of T-cell numbers, BTKi still indirectly impairs T-cell responses, while also causing damage to antitumor surveillance pathways.^[18–20]^ The local tumor microenvironment also plays a crucial role. BTKi impairs macrophage function and reprograms tumor-associated macrophages from a tumoricidal type 1 macrophage (M1) to a tumor-infiltrating M2 phenotype.^[21]^ This shift was associated with CD4 + T cells down-regulating interferon-γ (IFN-γ) and IL-2 and increasing IL-4 and IL-5 levels, cytokines that can promote tumor progression and suppress T helper 1 (Th1)-type responses characterized by IFN-γ production.^[22]^ Orelabrutinib may induce this myeloid environment in the skin, creating a local niche that allows multiple skin tumors to develop and grow in synergy with systemic immune changes.

Environmental exposures such as Ultraviolet irradiation had little relationship with SPM, and no antitumor immune response was seen in a phase II single-arm clinical trial evaluating Ibrutinib in melanoma cells, further suggesting that the multiple skin tumors of the patient in this case may be related to Orelabrutinib.^[20,23]^

Ibrutinib is the first-generation BTKi, although it is effective in the treatment of MCL, it is still discontinued or reduced due to significant side effects.^[4]^ Because of its structure, Ibrutinib binds to cysteine-utilizing kinases, such as epidermal growth factor receptor, steroid receptor coactivator kinase, B-lymphocyte kinase (BLK), tyrosine kinase (TEC), bone marrow tyrosine kinase gene (BMX), and ITK.^[24]^ Off-target effects of this irreversible BTKi on these kinases are thought to be associated with adverse drug reactions. The novel BTK inhibitor Orelabrutinib, developed by Chinese company InnoCare Pharma, was approved for market in China in December 2020 as a highly selective BTK inhibitor. It has shown better efficacy in the treatment of B-cell malignancies due to its higher selectivity and theoretically lower side effects associated with immunosuppression.^[25]^ The second-generation BTKi like Acalabrutinib, Zanubrutinib, and Orelabrutinib,^[26]^ have high efficiency and excellent kinase selectivity for BTK and minimize off-target inhibition of ITK or TEC and epidermal growth factor receptor family kinases.^[25]^

Clinically, most cutaneous BCC and SCC rarely spread or metastasized.^[27,28]^ In this case, surgical resection of the skin tumor was selected, the wound was repaired by using the adjacent skin flap on the nose and skin grafting on the chest, and the incision healed well. The patient and his family were satisfied with the results of the operation. No tumor recurrence or metastasis was found during follow-up, but the patient eventually died of a pulmonary infection and multiple organ failure. The cause of death was thought to be a consequence of immunosuppression, mainly related to MCL and its treatment, including Orelabrutinib. Although the skin tumor itself was not the direct cause of the patient’s death, the fatal lung infection occurred in a state of drug-induced immunosuppression.

We must acknowledge the limitations of this case report. Although the temporal correlation between the initiation of Orelabrutinib and the development of multiple skin tumors is noteworthy, we have no direct evidence of a causal relationship. Advanced patient age (77 years old) is also an important independent risk factor for BCC, SCC and AK, and the possibility that these benign and malignant tumors exist in a subclinical state or occur independently of drugs cannot be completely excluded. Therefore, patient functional analysis of immunity or staining for specific molecules (M1/M2 macrophage markers or regulatory T cell markers, for example, FOXP3) is direct evidence to confirm this immune dysregulation hypothesis.

Adverse effects associated with Ibrutinib typically develop during the first year of treatment. Although off-target effects were lower with Orelabrutinib, similar adverse effects were reported.^[29]^ Therefore It is recommended that MCL patients should check the skin status, such as the change of skin color and the presence of plaques, every 3 months during the use of BTK inhibitors. If patients have new pigmented spots or masses on the skin, oozing blood, exudation, or malodor secretion, they need to seek medical attention immediately. Dermoscopy is used for the initial evaluation of skin lesions that are suspected to be malignant. Reflectance confocal microscopy can be used for 3-dimensional imaging of lesions with irregular shapes or suspected malignancy. B ultrasound or CT can be used to assess the size and depth of invasion of the lesion when large lesions are detected or metastatic tumors are suspected. Early pathological biopsy was performed to determine the nature of the lesion. For patients in remote areas or with limited mobility, remote consultation can be achieved by transmitting images of skin lesions through smartphones or portable dermoscopy.

In conclusion, there may be a potential association between NMSC in MCL patients treated with BTKi and profound B-cell depletion, functional alterations of T cells, and reprogrammed tumor-promoting microenvironment, but further confirmation is needed. In the future, it is necessary to further accumulate Orelabrutinib-related adverse reaction data to optimize MCL treatment strategies.

## Acknowledgments

This work was supported by the second batch of major (key) science and technology plan projects in Jinhua (2022-3-101).

## Author contributions

**Funding acquisition:** Xuefeng Fu, Xuefeng Fu.

**Investigation:** Xuefeng Fu, Lei Zeng, Qiying Zhang, Xuefeng Fu.

**Writing – original draft:** Xuefeng Fu, Yuxi Zhang, Xuefeng Fu.

**Writing – review & editing:** Xuefeng Fu, Yuxi Zhang, Xuefeng Fu.
